# Comparing Decentralized Learning Methods for Health Data Models to Nondecentralized Alternatives: Protocol for a Systematic Review

**DOI:** 10.2196/45823

**Published:** 2023-06-19

**Authors:** José Miguel Diniz, Henrique Vasconcelos, Júlio Souza, Rita Rb-Silva, Carolina Ameijeiras-Rodriguez, Alberto Freitas

**Affiliations:** 1 CINTESIS—Centre for Health Technology and Services Research Faculty of Medicine University of Porto Porto Portugal; 2 PhD Program in Health Data Science Faculty of Medicine University of Porto Porto Portugal; 3 MEDCIDS—Department of Community Medicine, Information and Health Decision Sciences Faculty of Medicine University of Porto Porto Portugal

**Keywords:** decentralized learning, distributed learning, federated learning, centralized learning, privacy, health, health data, secondary data use, health data model, blockchain, health care, data science

## Abstract

**Background:**

Considering the soaring health-related costs directed toward a growing, aging, and comorbid population, the health sector needs effective data-driven interventions while managing rising care costs. While health interventions using data mining have become more robust and adopted, they often demand high-quality big data. However, growing privacy concerns have hindered large-scale data sharing. In parallel, recently introduced legal instruments require complex implementations, especially when it comes to biomedical data. New privacy-preserving technologies, such as decentralized learning, make it possible to create health models without mobilizing data sets by using distributed computation principles. Several multinational partnerships, including a recent agreement between the United States and the European Union, are adopting these techniques for next-generation data science. While these approaches are promising, there is no clear and robust evidence synthesis of health care applications.

**Objective:**

The main aim is to compare the performance among health data models (eg, automated diagnosis and mortality prediction) developed using decentralized learning approaches (eg, federated and blockchain) to those using centralized or local methods. Secondary aims are comparing the privacy compromise and resource use among model architectures.

**Methods:**

We will conduct a systematic review using the first-ever registered research protocol for this topic following a robust search methodology, including several biomedical and computational databases. This work will compare health data models differing in development architecture, grouping them according to their clinical applications. For reporting purposes, a PRISMA (Preferred Reporting Items for Systematic Reviews and Meta-Analyses) 2020 flow diagram will be presented. CHARMS (Critical Appraisal and Data Extraction for Systematic Reviews of Prediction Modelling Studies)–based forms will be used for data extraction and to assess the risk of bias, alongside PROBAST (Prediction Model Risk of Bias Assessment Tool). All effect measures in the original studies will be reported.

**Results:**

The queries and data extractions are expected to start on February 28, 2023, and end by July 31, 2023. The research protocol was registered with PROSPERO, under the number 393126, on February 3, 2023. With this protocol, we detail how we will conduct the systematic review. With that study, we aim to summarize the progress and findings from state-of-the-art decentralized learning models in health care in comparison to their local and centralized counterparts. Results are expected to clarify the consensuses and heterogeneities reported and help guide the research and development of new robust and sustainable applications to address the health data privacy problem, with applicability in real-world settings.

**Conclusions:**

We expect to clearly present the status quo of these privacy-preserving technologies in health care. With this robust synthesis of the currently available scientific evidence, the review will inform health technology assessment and evidence-based decisions, from health professionals, data scientists, and policy makers alike. Importantly, it should also guide the development and application of new tools in service of patients’ privacy and future research.

**Trial Registration:**

PROSPERO 393126; https://www.crd.york.ac.uk/prospero/display_record.php?RecordID=393126

**International Registered Report Identifier (IRRID):**

PRR1-10.2196/45823

## Introduction

### Background

The current health paradigm challenges are unprecedented in their nature and scope. Stemming from a growing [1], aging [1], and comorbid [2] global population, disability is becoming an increasingly large share of the burden of disease and of the already unsustainable health care costs [3-6]. Faced with the need to invest in more effective and preventive strategies, improved research and development are essential to address these unwavering issues [2].

To do so, robust evidence-based health knowledge is needed, as a way of enabling improved planning and provision of care. Presently, many data-driven approaches are commonplace in biomedical sciences and clinical research. These include examples from epidemiological surveillance [7] and cancer prognosis [8] to drug discovery [9,10] and mortality prediction [11].

Thus, access to data has been the foundation for better health models, both in their precision and validity to represent different medical conditions [12,13] and patients [14,15]. In parallel, alongside recent digital transitions and new tools and infrastructures, data analysis has become more powerful and faster than ever [16]. This gave rise to data science [17], resulting from the complex merger of traditional statistics disciplines combined with other subjects.

In particular, data mining techniques have elevated the computational functionality, especially for cognitive and analytic processes that are hard to develop algorithmically [18]. Thus, machine learning models, such as decision trees, linear regression, and support vector machines are now abundant in health research [19,20]. Deep neural networks brought to light even more sophisticated applications of natural language processing and computer vision, both effective and powerful [21,22].

Supported by robust software and hardware, these new model development approaches rely on the availability of large and high-quality training databases. Hence, the concept of big data emerged, referring to the need for comprehensive data sets to sustain the inferential process in both their internal and external validities. While it is often characterized by a few key dimensions (volume, variety, velocity, value, veracity, and variability), many other features (eg, venue and volatility) can be of relevance [23,24]. However, concerns regarding privacy protection—a fundamental human right [25]—are rising amid increasing numbers of misconducts and violations [26,27].

### Limitations of Current Strategies

In response to both data demands and privacy challenges, 2 groups of arguments can be made in favor of transitioning traditional approaches toward a new data science paradigm.

First, to generate and use high-quality big data to support precision and generalizability assumptions, findable, accessible, interoperable, and reusable (FAIR) principles [28] ought to be adopted. In theory, following these criteria is important to develop new scientific studies and validate, or otherwise reproduce, at least part of already available works [29,30]. In practice, FAIR principles are hard to comply with, for several reasons.

Starting with “findable,” few health data catalogs are available, and some are no longer being updated [31-33]. Considering access, while new instruments [34-37] have presented legislation and guiding frameworks on how data should be used and shared, their impact is controversial and their implementation is often complex, especially for medical research purposes [38-44]. Even follow-up developments, like the European Health Data Space [45], are viewed with skepticism by some member states regarding its practical feasibility and results [46,47].

Furthermore, data use is often limited by its interoperable characteristics. Technical heterogeneity due to different electronic health record systems, data standards, and data exchange protocols makes it difficult to share and integrate health data across multiple parties. Given all the challenges stated, reusability is practically impossible, outside some rare contexts [48,49].

Additionally, another set of arguments can be presented referring to the need for a systematic approach that does not rely on individuals’ actions nor benefits from their limitations. First, we recognize that individuals are not capable of always acting with their own (or collective) interests in mind [50]. Specifically in their health data sharing attitudes, even though people may be aware of the value of data and potential privacy issues, their actions are often contrasting to their stated beliefs [51,52].

Then, we place the onus and burden of sustainable privacy protection on the system itself and are not reliant on the constant and best behavior of every individual agent [53]. Some successful global data sharing efforts [48,54,55] have already proven that after the demanding set up, it is possible to not only improve access to data but also protect privacy by design [56].

### New Technological Solutions

In response to these demands, recent breakthroughs in some computational domains, including secure processing units [57,58], differential privacy [59,60], and homomorphic encryption [61,62], have offered technical alternatives for the use of data in a privacy-preserving fashion. Being one of the more interesting approaches, decentralized learning architectures [63,64] enable data scattered across different silos (eg, health care providers) to be used to develop or validate pre-existing models. By combining the information derived from data present in each silo, it is possible to create more precise and generalizable models.

In general, local models are first developed using the party’s own data. Second, only the model parameters (ie, information) are shared, usually with a central coordinator, responsible for aggregating the different local instances to create a new decentralized model. Throughout this process, data remain unmoved and are not accessed or manipulated by third parties.

Accordingly, such models can be produced without mobilizing or otherwise sharing the data set itself by parceling out the inferential process, on top of distributed computation principles [65]. By cyclically repeating this process, we can improve the model performance and include newly available data, fostering the development of continuously updated real-world evidence-based knowledge.

The potential for developing health care applications and generating value is clear [66]. Sharing models developed using different data sets can make the distributed solutions more comprehensive and adequate, due to robust internal and external validations. These approaches are useful to study medical conditions [67-69], especially those with limited prevalence or few observations, and prevent inadequate care due to mis- or underrepresentation of certain groups of patients [70].

Subsequently, significant agreements, like the one achieved between the United States and the European Union [71], promise a new platform for these technologies to be implemented while respecting their differing legislative frameworks. Such consensus can have a seismic impact on the way data science is conducted.

In the meantime, there still are many ongoing challenges regarding decentralized learning [18,72]. Some relevant issues are a lack of objective and measurable standard definitions of privacy and security [73], and server-client trust and honesty assumptions to computationally intensive and energetically demanding tasks [61,74]. Other important problems are the heterogeneity in distributed data and environments as well as fairness and respect for individual and local preferences [73].

However, above all else, the most pressing undertaking remains assessing the validity, relevance, and applicability of already published and available tools to inform and justify subsequent health technological appraisals and their implementation in real-world settings.

To do so, we must address the following questions: are decentralized health data models’ performance superior (or noninferior) to current (centralized and local) approaches? and what are the reported privacy gains and the main resource demands?

### Aims and Objectives

The systematic review based on this protocol aims to, first, compare the performance among health data models developed or validated using decentralized learning approaches (eg, federated and blockchain) to those developed using nondecentralized methods (eg, centralized and local). These can include applications such as automated diagnosis, segmentation of lesions or features, as well as mortality prediction.

The performance metrics used for model comparison will be the following: area under the receiver operating characteristic (AUROC) curve, *F*_1_ score, Jensen-Shannon distance, sensitivity (or recall), specificity, accuracy, precision (or positive predictive value), negative predictive value, Dice score, as well as any metrics regarding the convergence step. Comparisons will be made only among models using the same type of data (eg, tabular and images) and clinical application (eg, diagnostic and survival). Our secondary aims are to compare the privacy compromise (eg, privacy budget) and resource use (eg, computation power and wall-time) among these health data models using different architectures.

With this review study, our goal is to summarize the progress and findings from state-of-the-art decentralized learning models in health care, in comparison to their currently used counterparts. These results are expected to clarify the consensuses and heterogeneities reported and help guide the research and development of new robust and sustainable applications to address the health data privacy problem, with applicability in real-world (clinical) settings.

## Methods

### Eligibility Criteria

Regarding the inclusion criteria, relevant studies are original research papers (including published, unpublished, and preprints) targeting one or multiple specific human medical conditions. They should be comparing 2 types of health model learning approaches—one decentralized (eg, federated and blockchain) and other nondecentralized (eg, centralized and local). For this review, decentralized learning architectures are defined as a machine learning approach to use data available from multiple parties, without sharing them with a single entity, to extract information [18].

As there may be some confusion regarding the terms “decentralized” and “distributed,” we consider the first as the most appropriate and rigorous designation of our study field, including federated and blockchain architectures. The latter is broader in scope and refers to a computational subject that includes, but also precedes, the current advances and innovations [65]. Nevertheless, studies will be included regardless of the adopted terminology if the definition or methodologies used are in accordance with the above definition of decentralized learning architectures. Both types of models must report at least one of the following model performance metrics: AUROC curve, *F*_1_ score, Jensen-Shannon distance, sensitivity (or recall), specificity, accuracy, precision (or positive predictive value), negative predictive value, Dice score, as well as any metrics regarding the convergence step.

Regarding the exclusion criteria, papers published before 2012 will not be considered for this analysis, due to the following reasons. First, the earliest decentralized learning framework proposals in health care are only introduced [75,76] or applied [77] after this year. Second, seminal studies detailing and developing the current definition of these concepts were published in 2016 [78,79], with related works being as available as early 2014 [63,80,81]. Moreover, in no systematic review for health care implementations are there primary studies published before 2016 [82-86].

Each synthesis will consider studies with common types of clinical applications (intervention, diagnosis, etiology, prevention, prognosis or prediction, quality of life or meaning, and therapy), types of models, and types of data used. Whenever possible, they will be grouped by health problems.

### Information Sources

Recognizing the interdisciplinarity nature of the research being made on health data models, several databases will be queried—some more specific to biomedical scientific research (ie, PubMed, SpringerLink, and Lippincott Williams & Wilkins), some more specific to computer science and informatics engineering (ie, the Association for Computing and Machinery Digital Library or Guide to Computing Literature and IEEE Xplore), while others were more general (ie, Wiley Online Library, Scopus, Web of Science, and Lens).

Moreover, 2 registries for systematic reviews (Cochrane Database of Systematic Reviews and PROSPERO) were surveyed for submissions related to these topics, in order to look for additional primary papers. Furthermore, queries were also conducted in databases, which included research papers not peer-reviewed or otherwise unpublished (eg, medRxiv and arXiv). For every listed source, searches are expected to be conducted during March 2023.

Five experts in relevant scientific fields (from data science, health informatics, and decentralized learning approaches) will be contacted to ask for suggestions of additional bibliography not included in the selection process, without the knowledge of the selected or rejected papers. Such recommendations will be considered worthy of consideration and included if eligibility criteria are met. Due to the reasons stated in the eligibility criteria, it was deemed appropriate to restrict the search to papers published in 2012 or later.

### Search Strategy

#### Overview

Given the recency of this research domain and the expected limited number papers, it was imperative to devise a broad search strategy. This was materialized in choices such as including several databases, as well as using synonyms and wildcards in the query.

However, due to the popularity of some of the query terms—for example, distributed, model, training, and health—some procedures were adopted to filter noise. For instance, words like “distributed” and “model” should have a limited number of words in between, for the finding to be relevant. Moreover, search engines have heterogeneous features, which make it difficult to conduct the desired exploration.

**Figure 1:**
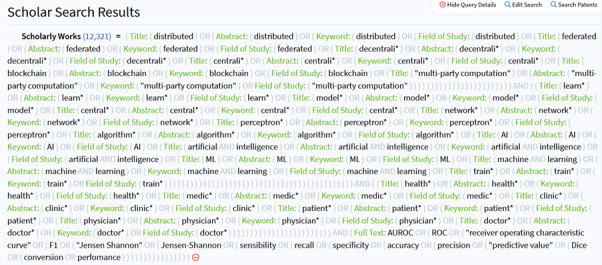
Example of search query as used on Lens database. AI: artificial intelligence; AUROC: area under the receiver operating characteristic; ML: machine learning; ROC: receiver operating characteristic.

Hence, a composite search strategy was adopted. The first part, optimizing for comprehensiveness, was focused on writing the query and electing relevant filters for each database used (see “Part 1—Database Query” section, Table 1, and Figure 1). Subsequently, a filtration process was applied, using regular expressions (RegEx) code, to make up for the lacking features of the databases used—such as word proximity limits, operators, and metadata fields searched (see “Part 2—Results Filtration” section).

**Table 1:** General query terms by group.

Group	Terms
A—Model architecture	decentrali*, distributed, federated, central*, multi-party computation, blockchain
B—Model synonym	learn*, model*, network*, AI^a^, artificial intelligence, ML^b^, machine learning, train*, tensor*, perceptron, algorithm*
C—Health related	health*, medic*, clinic*, patient*, physician*, doctor*
D—Performance metrics	AUROC^c^, ROC^d^, receiver operating characteristic curve, *F*_1_, Jensen-Shannon, sensitivity, recall, specificity, accuracy, precision, predictive value, Dice, conversion

^a^AI: artificial intelligence.

^b^ML: machine learning.

^c^AUROC: area under the receiver operating characteristic.

^d^ROC: receiver operating characteristic.

#### Part 1—Database Query

First, a simpler version of the query, suitable for all search engines, was used to retrieve a less specific group of abstracts.

For groups A, B, and C, the fields Title, Abstract, Keyword, and Field of Study, when available, will be searched. Terms from group A and B must be near each other, with a maximum of 2 words in between them. For terms in group D, the full-text document will be searched. The query will not be case-sensitive. The * symbol represents the wild card.

As per eligibility criteria, only primary papers from 2012 and beyond will be relevant. Thus, the search query will look for papers with at least 1 term, within the considered fields, from every group.

For each source, a specific query will be produced. Documentation will be made available providing the exact search string, a URL (if possible), and other details, such as filters applied.

#### Part 2—Results Filtration

As some indispensable terms are very prevalent in publications, such as “model” and “distribution,” increasing the relative useful yield of the query and the number of studies retrieved, we conducted a processing task to filter irrelevant studies.

To do so, using RegEx code in R (R Foundation for Statistical Computing), we simulated a “within” operator. It was developed to only capture studies in which the term referring to the model architecture (group A) and the one referring to the model synonym (group B) have no more than 2 other terms separating them. Finally, while this process does not perfectly compensate for the limitation in the databases search features and variation, it is expected to offset the most significant differences and not significantly compromise the pursuit of relevant primary papers.

### Selection Process

The selection of the primary studies will comprise 2 moments: the screening phase—when the papers are appraised using only their title and abstract, and the inclusion decision phase—when the papers are appraised using their full-text versions. To manage the appraisal of the retrieved primary studies, the Rayyan [87] software suit (Rayyan) will be used. Before the screening phase, exact matches and additional duplicates will be removed, quantifying the number of papers ruled out.

All papers retrieved through the application of the search methods detailed above will be screened using their title and abstract by 3 researchers acting independently and blinded to each other’s decisions. Excluded papers should be labeled using the first unmet criteria of the inclusion criteria.

When there is not a complete agreement on the inclusion (or exclusion) decision, the evaluating researchers will discuss and attempt to achieve a consensus, with potential consultation with the other authors. If that is not possible, the majority decision will be chosen.

After the screening phase, the same sequence of study appraisal and disagreement resolution will be conducted for the full-text versions of the papers. The flow of papers included and excluded will be represented in a diagram, where quantity, source (search method and database), and reason for decision will be explicit.

The flow diagrams for the papers included and excluded will be represented according to the PRISMA (Preferred Reporting Items for Systematic Reviews and Meta-Analyses) 2020 guidelines. In the end, a final list of all the primary studies selected to be included in the review will be presented with a complete reference and, when possible, a DOI link.

### Data Collection Process

For each study, data extraction will be conducted by 3 researchers, who will work independently, in a blinded fashion, by reading the full-text versions (or other versions if full text is not available or does not exist). They will use custom-made web-based forms, including the CHARMS (Critical Appraisal and Data Extraction for Systematic Reviews of Prediction Modelling Studies) checklist items for reporting quality and risk of bias assessment [88]. These forms will be piloted before the data collection process.

After completion, retrieved data will be compared to check for errors or inconsistencies and discuss any doubts. The researchers will decide by consensus on the last version of the database of the data collected.

### Data Items

A pilot study was conducted to refine the list of relevant data variables to collect, using 100 papers of a developing query. The complete list of variables for collection (whenever available) is the following.

First, general attributes are to be collected, namely, title, author or authors, abstract, publication date, country or countries of the research institutions, type of publication, the journal or publisher, as well as the PICO (Population, Intervention, Comparison, and Outcomes) question.

Specific data points regarding health topics, the type of clinical application (eg, diagnostic and survival), and the applicable medical domain will also be registered.

Concerning the data used, information about the data set size, namely, the number of observations or cases, the number of variables, and the volume in megabytes or gigabytes will be detailed. As far as the nature of the data goes, it will be marked as either synthetic or real, and, in the latter, whether the data were collected for the study (primary source) or not (secondary source). The original data holders will be described by their number, type, and localization, as well as the storage architecture used (eg, centralized, decentralized, and local). Other data points will be the places (geographical and institutional) where data were analyzed, the ethics and legal permissions reported, as well as the data type used (eg, text and images), alongside their format (eg, tabular), and conversion processes.

The type of all models reported are, for example, deep neural network and decision trees, and their performance metrics are specifically AUROC curve, *F*_1_ score, Jensen-Shannon distance, sensitivity or recall, specificity, accuracy, precision or positive predictive value, negative predictive value, Dice score, as well as any metrics regarding the convergence step. The training methods will also be registered, including their update routine (rounds, epochs, quorum, update frequency, update content, and protocols for data communication) and statistical methods used. The validation process used will also be described.

Regarding the architectures used, their type, data flow, and client type (cross-silo vs cross-device vs both) will be extracted, as well as the personalization or customization step and the aggregation methods used. Importantly, we will detail all the model architectures and types compared, and their hypothesis tests.

For our secondary aim, we will collect data on the privacy cost measures (privacy budget, k-anonymity, fingerprinting, entropy, or others) and resource consumption (from computer resources, hardware and software specs, and energy to time and number of rounds), as well as additional security and privacy protection measures.

Lastly, we will record the reason for using decentralized approaches, whether the data and code used are available, and the reported challenges and limitations. No assumptions will be made regarding missing or unclear information—those findings will be reported as such.

### Study Risk of Bias Assessment

For each selected paper, the CHARMS [88] and PROBAST (Prediction Model Risk of Bias Assessment Tool) [89] checklists will be used to assess the risk of bias of included research works. The full results of such an appraisal will be presented on a table and considered for the discussion of the results. If deemed relevant, depending on the final list of results, other more specific tools may be used.

### Effect Measures

All the effect measures in the original studies will be presented. Whenever unavailable, and if possible, the difference between the AUROC curve estimates for the models of different architectures will be calculated. When multiple rounds of model development, validation, or application exist, the difference in time and rounds to a set performance target will be calculated.

If multiple values for each model are available, the median and mean values will also be used to calculate the differences. If AUROC curve values are not available, other commonly found metrics may also be used.

### Synthesis Methods

A qualitative analysis of the evidence will be conducted alongside a descriptive synthesis of the results. Each synthesis will consider the types of clinical applications, models, and data used. Whenever possible they will be grouped by health problems.

Missing data will be reported as “Missing.” Whenever a synthesis method is not applicable, it will be labeled as “Not Applicable.”

Due to the heterogeneity of the applications and model characteristics, it is not expected to be able to synthesize results in a quantitative fashion. Accordingly, neither a meta-analysis nor a sensitivity analysis will be performed.

### Reporting Bias Assessment

For every eligible study, the authors will look up preresearch registers of protocols and check for differences in the published work. Additionally, they will assess whether any pertinent statistics or analyses are unreported. All corresponding authors will be contacted to assess if they have any nonreporting experience with any version of their published work or regarding other unpublished research, as suggested by Ammenwerth and de Keizer [90].

Questions may include the following: Which information systems did you evaluate in the last 3 years? Where did you publish the results? and If you did not publish them, what were the reasons for that decision? (here, some common reasons could be selected or added using free text fields).

Moreover, specific efforts will be made to identify omissions of some measured outcomes, as well as selective reporting of only “significant” findings from among several analyses undertaken.

### Certainty Assessment

Certainty assessment procedures will be performed if appropriate instruments are available at the time of review completion.

### Other Information

Efforts will be made to make available the query links (or prompts) for each database used, as well as the RegEx filtering code, the templates for data collection forms, and the data extracted from the included studies, and any other resources, which might be used for the subsequent review.

## Results

The queries and data extractions are expected to start on February 28, 2023, and end by July 31, 2023. The research protocol was registered with PROSPERO, under the number 393126, on February 3, 2023.

It is expected that the systematic review will summarize the progress and findings from state-of-the-art decentralized learning models in health care, in comparison to their local and centralized counterparts. These results will help in clarifying the consensus and heterogeneities reported among different models and studies, as well as guide the research and development of new robust and sustainable applications to address the health data privacy problem, with applicability in real-world (clinical) settings.

## Discussion

### Principal Findings

As the systematic review is yet to be conducted, no specific results can be reported at this time. However, this will be the first systematic review on the comparison of decentralized health data models to more common local or centralized approaches that is both comprehensive and focused on objective performance metrics. Due to our exhaustive search strategy and the plurality of data sources identified, this work will present the clearest situational assessment yet of the application of these technologies in health care.

We hope that, by highlighting effective models and their developments, as well as identifying those which underperformed, we can shed light on more fruitful and interesting directions for our peers. In addition, by considering the development costs and privacy gains, as reported in the primary papers, it is expected that this review may be useful for health technology assessment and evidence-based decisions, from health professionals, data scientists, and policy makers alike.

By providing the original search queries and documentation on the methods used, we open the possibility for researchers to use this protocol and upcoming supplemental materials to not only audit and validate our work but also conduct updated versions of this review as new evidence is published.

### Strengths and Limitations

While decentralized health data models are a nascent and growing field of research, all appropriate steps will be taken to ensure a comprehensive and exhaustive search of available literature. To do so, we will consider a variety of sources (both specific to bio- and computational sciences and generalist databases), as well as preprint works. Alongside these efforts, a substantive yet clear search and selection procedures have been detailed, with 3 authors selecting primary papers for inclusion in the review. Together, these will also increase the sensitivity and specificity of the search results and the studies included for analysis.

Moreover, this protocol is already registered with PROSPERO and is written in accordance with the PRISMA 2020 guidelines, which will confer more validity and accountability for the results provided. In conjunction with the review, these actions will allow for reproducible and auditable work. For instance, it may be useful, with the necessary adaptations, to repeat this study, when even more evidence is available.

Some limitations of this work include the expected heterogeneity among primary studies, complicating synthesis and comparability among models, and the rapid evolution of the field. Given the unusual population (the P in PICO) of this systematic review, the appraisal of the primary studies may be made difficult by a lack of appropriate tools to assess the risk of bias, especially in reporting, and the certainty of the findings.

### Comparison With Prior Work

While there are some systematic reviews on the topic already available, they present several important shortcomings in the size and scope [82-84], the specificity of health care applications [85], and the capability and comprehensiveness of query prompts [86]. Moreover, to the best of our knowledge, none of them were accompanied by a protocol publication or registry before the corresponding review.

Therefore, a more robust and valid synthesis of the currently available scientific evidence must be conducted. We consider the proposed systematic review is capable of achieving that result.

### Conclusions

Increasing pressure to develop better, more effective, and cost sensitive care, juxtaposed with the privacy-preserving principles and methodologies, has created a considerable demand for alternative health data model development, validation, and application procedures.

While decentralized approaches, such as those built with federated and blockchain architectures, promise considerable gains for extracting information out of health data, there is much uncertainty regarding how they compare to current centralized and local models, as well as their associated privacy gains and their resource consumptions.

This protocol is the first, at the time of this writing, to outline a systematic review on health data decentralized models, aiming not only to capture the rich variety and complexity of available research but also to generate a rigorous and comprehensive assessment of the synthesis of their results and conclusions.

It is expected that such work will have implications for this budding research field and policy making, especially for those working with health data privacy matters. This review will highlight the advances and shortcomings of these approaches to better inform the development and application of new tools in service of patients’ privacy while hoping to guide future research.

## Abbreviations

AUROC: area under the receiver operating characteristic
CHARMS: Critical Appraisal and Data Extraction for Systematic Reviews of Prediction Modelling Studies
FAIR: findable, accessible, interoperable, and reusable
PICO: Population, Intervention, Comparison, and Outcomes
PRISMA: Preferred Reporting Items for Systematic Reviews and Meta-Analyses
PROBAST: Prediction Model Risk of Bias Assessment Tool
RegEx: regular expression

## Data Availability

Documentation will be provided regarding the specific queries used, tailored to each source, including their adapted formulation and filters, to ease reproducibility. Whenever possible, a direct URL link will be included. In addition, the code used to compile and filter the results of the various extraction files will be freely available in a GitHub repository. The original lists of all papers (as extracted) as well as other reports with all those subjected to the screening process will be accessible in the same repository. Moreover, the original data collected from each of the studies included in the review will be shared in a Research Data Repository.
